# Regional Coherence Alterations Revealed by Resting-State fMRI in Post-Stroke Patients with Cognitive Dysfunction

**DOI:** 10.1371/journal.pone.0159574

**Published:** 2016-07-25

**Authors:** Cheng-Yu Peng, Yu-Chen Chen, Ying Cui, Deng-Ling Zhao, Yun Jiao, Tian-Yu Tang, Shenghong Ju, Gao-Jun Teng

**Affiliations:** 1 Jiangsu Key Laboratory of Molecular and Functional Imaging, Medical School of Southeast University, Nanjing, Jiangsu, China; 2 Department of Radiology, Zhongda Hospital, Medical School of Southeast University, Nanjing, Jiangsu, China; 3 Department of Radiology, Nanjing First Hospital, Nanjing Medical University, Nanjing, China; Institute of Psychology, Chinese Academy of Sciences, CHINA

## Abstract

**Objectives:**

Post-stroke cognitive dysfunction greatly influences patients’ quality of life after stroke. However, its neurophysiological basis remains unknown. This study utilized resting-state functional magnetic resonance imaging (fMRI) to investigate the alterations in regional coherence in patients after subcortical stroke.

**Methods:**

Resting-state fMRI measurements were acquired from 16 post-stroke patients with poor cognitive function (PSPC), 16 post-stroke patients with good cognitive function (PSGC) and 30 well-matched healthy controls (HC). Regional homogeneity (ReHo) was used to detect alterations in regional coherence. Abnormalities in regional coherence correlated with scores on neuropsychological scales.

**Results:**

Compared to the HC and the PSGC, the PSPC showed remarkably decreased ReHo in the bilateral anterior cingulate cortex and the left posterior cingulate cortex/precuneus. ReHo in the bilateral anterior cingulate cortex positively correlated with the scores on the Symbol Digit Modalities Test (*r* = 0.399, *P* = 0.036) and the Complex Figure Test-delayed recall subtest (*r* = 0.397, *P* = 0.036) in all post-stroke patients. Moreover, ReHo in the left posterior cingulate cortex/precuneus positively correlated with the scores on the Forward Digit Span Test (*r* = 0.485, *P* = 0.009) in all post-stroke patients.

**Conclusions:**

Aberrant regional coherence was observed in the anterior and posterior cingulate cortices in post-stroke patients with cognitive dysfunction. ReHo could represent a promising indicator of neurobiological deficiencies in post-stroke patients.

## Introduction

Human society attaches great importance to stroke due to its high incidences of lethality and disability [1]. The leading causes of stroke-induced disability include not only movement disorders but also cognitive dysfunction. Nearly 35% of all ischaemic stroke patients have slight to moderate levels of cognitive impairment [2], and 25% of all post-stroke patients develop dementia within 15 months [3–6]. These disorders impact patient quality of life and consume large amounts of health resources [1]. Potential neuro-pathophysiological mechanisms of post-stroke cognitive dysfunction have been proposed. First, the large vascular obstruction could directly cause avascular necrosis of a major functional unit within the cerebral cortex [7]. Alternatively, the abnormal production and delivery of neurotransmitters such as dopamine and acetylcholine after stroke might induce damage to neural circuits associated with cognition [8, 9]. The demyelination of secondary white matter and the deposition of amyloid β-protein due to ischaemia could also result in post-stroke cognitive decline [10]. Nonetheless, the exact mechanisms responsible for post-stroke cognitive dysfunction remain unclear.

Neuroimaging is the technique most commonly used to assess cerebral diseases [11]. Functional magnetic resonance imaging (fMRI), especially resting-state fMRI, has been established to be a useful noninvasive technique for determining how structurally segregated and functionally specialized cerebral centres are interconnected; these relationships are reflected by fluctuations in low-frequency (0.01–0.1Hz) blood oxygenation level-dependent (BOLD) signals [12–14]. Previous studies have employed resting-state fMRI to examine multiple whole-brain networks, especially the default-mode network (DMN), to explore the neural changes occurring in stroke patients [15–20]. Tuladhar et al. found decreased intra-network functional connectivity (FC) in the left medial temporal lobe, the posterior cingulate cortex (PCC) and the medial prefrontal cortex within the DMN and reduced inter-network FC among these regions in stroke patients [15]. Wang et al. confirmed that stroke patients exhibited decreased intra-network FC within the anterior DMN [16]. By contrast, Dacosta-Aguayo et al. found that stroke patients showed increased DMN activity, which was more widespread in the left precuneus and the left anterior cingulate cortex (ACC) [17, 18]. Furthermore, Park et al. found that the increased FC of the contralesional dorsolateral prefrontal cortex within the DMN positively correlated with cognitive functional recovery in stroke patients, and this alteration might be considered as a compensatory mechanism for overcoming cognitive impairment [19]. Together, these results suggest that the DMN may play a pivotal role in the neurological pathophysiology of post-stroke cognitive dysfunction.

In contrast to FC, regional homogeneity (ReHo), a whole-brain resting-state fMRI parameter, can be used to identify aberrant coherence of local neural activity across the entire brain [21]. Aberrant ReHo may be linked to the disequilibrium of spontaneous neural activity within and between corresponding brain regions; this disequilibrium has been related to several neurological impairments, such as mild cognitive impairment [22], Alzheimer’s disease [23] and transient ischaemic attack [24]. ReHo could be used to map local spontaneous neural activity, also referred to as short-term connectivity, while FC could be used to map long-term connectivity. Evaluation of short-term connectivity can provide information that evaluation of long-term connectivity cannot. For example, if the FC between a certain seed area and other brain regions is reduced, it is necessary to determine whether the reduced FC between the seed region and other brain regions reflects neural damage or simply a decrease in the local spontaneous neural activity of one of the involved brain regions [25]. ReHo can be used to detect undiscovered haemodynamic responses that FC cannot reveal [21]. Thus, ReHo is a potentially powerful tool for investigations the alterations in resting-state brain activity, thereby complementing the information provided by FC analysis.

Based on prior work and theoretical considerations, we aimed to explore the alterations in regional coherence in patients with cognitive dysfunction following subcortical ischaemic stroke using a ReHo algorithm. We presumed that (1) abnormal regional coherence reflected by ReHo occurs within brain regions related to the DMN in post-stroke patients with cognitive dysfunction and correlates with deficiencies in specific cognitive domains and that (2) post-stroke patients with poor cognitive dysfunction (PSPC) show different ReHo patterns from post-stroke patients with good cognitive function (PSGC).

## Materials and Methods

### Subjects

Ethical approval of this study was provided by Institutional Review Board of Zhongda Hospital affiliated to Southeast University. Written informed consent was obtained from all participants. We recruited 32 subcortical stroke patients (10 females and 22 males; age range: 45 to 75 years, 59.95 ± 7.95 years) from the Neurological Department of Zhongda Hospital from December 2013 to July 2015. In addition, thirty healthy subjects (13 females and 17 males; age range: 48 to 69 years, 56.97 ± 6.25 years) were also recruited through advertisements and were matched to the post-stroke patients with respect to age, sex and education. The inclusion criteria for the post-stroke patients were as follows: (1) between 45 and 75 years old, (2) right-handedness before stroke; (3) first-ever subcortical ischaemic stroke; (4) time after stroke onset ≥ 3 months, as 3 months was previously identified as the first time point of long-term changes related to cognitive function [6, 26, 27]; and (5) absence of serious movement disorders (whole-extremity Fugl-Meyer Assessment score > 90 and Barthel Index > 90). The exclusion criteria for both post-stroke patients and healthy controls (HC) were as follows: (1) any neuropsychiatric comorbidity such as depression; (2) any clinically significant or unstable medical disorder; (3) severe white matter hyperintensity manifesting as a Fazekas [28] scale score > 1; or (4) any contraindication for MRI.

### Clinical Data and Neuropsychological Tests

The clinical data, including cardiovascular disease risk factors, Fugl-Meyer Assessment scores and Barthel Indexes, were obtained from questionnaires and medical records. The cognitive performance of each participant was tested using a battery of neuropsychological tests related to various cognitive domains. The Mini-Mental State Examination (MMSE) [29] and the Montreal Cognitive Assessment (MoCA) [30] were used to assess general cognitive function. The Auditory Verbal Learning Test (AVLT), the Complex Figure Test (CFT) and their delayed recall subtests were used to assess episodic verbal and visual memory. The Forward Digit Span Test (DST-F) [31] and the Symbol Digit Modalities Test (SDMT) [32] were used to assess attention abilities. Visuo-motor coordination and speed were assessed using the Trail Making Test Part A (TMT-A) as well as the SDMT. Executive abilities were explored using the Backward Digit Span Test (DST-B) [31] and the Trail Making Test Part B (TMT-B). Each subject required 70 minutes to complete the neuropsychological examination. Post-stroke patients were stratified into two subgroups (PSGC and PSPC) according to their MoCA scores, as the MoCA is widely used and recognized as one of the best screening tests for cognitive dysfunction [30]. In a previous study, an adjusted cut-off MoCA score of < 24 was recommended to identify patients with cognitive dysfunction [33]. Therefore, the PSGC subgroup was defined by a MoCA score of ≥ 24, and the PSPC subgroup was defined by a MoCA score of < 24.

### Brain MRI Data Acquisition

All imaging data were acquired using a 3.0-T MRI scanner (Siemens MAGENETOM Trio, Erlangen, Germany) equipped with a standard head coil at Zhongda Hospital, Nanjing, China. The subjects lay supine with their heads restrained by foam pads to reduce head motion and with earplugs in their ears to reduce perception of scanner noise. Functional images were obtained axially using a gradient-recalled echo-planar imaging (GRE-EPI) sequence according to the following scanning parameters: slices = 36; repetition time (TR) = 2,000 ms; echo time (TE) = 25 ms; thickness = 4 mm; gap = 0 mm; field of view = 240 × 240 mm; flip angle = 90°; and acquisition matrix = 64 × 64. All subjects were instructed to rest quietly with their eyes closed and to avoid thinking of anything specific during scanning. Structural images were acquired using a T1-weighted three-dimensional spoiled gradient-echo sequence according to the following scanning protocol: slices = 176; TR = 1,900 ms; TE = 2.48 ms; thickness = 1 mm; gap = 0 mm; flip angle = 90°; acquisition matrix = 256 × 256; and field of view = 250 × 250 mm. In addition, T1-weighted, T2-weighted and fluid-attenuated inversion recovery images were also acquired to detect ischaemic lesions.

### MRI Data Analysis

The preprocessing of the resting-state functional images and the ReHo analysis were conducted using the following two software tools: Data Processing Assistant for Resting-State fMRI (DPARSF; http://www.restfmri.net/forum/DPARSF) and Resting-State fMRI Data Analysis Toolkit (REST; http://www.restfmri.net); both tools are based on statistical parametric mapping (SPM8; http://www.fil.ion.ucl.ac.uk/spm). The first 10 volumes of each time series were deleted to account for the time required for the participants to adapt to the scanning. Then, slice timing and realignment for head-motion correction were performed on the remaining 230 images. The participants with head motion of > 2.0 mm in maximum displacement or > 2.0° rotation in angular motion were excluded from the study. The remaining dataset was spatially normalized to the Montreal Neurological Institute (MNI) EPI template (resampling voxel size = 3 × 3 × 3 mm^3^). Several sources of spurious variances, including the estimated motion parameters and the average time series in the cerebrospinal fluid and white matter regions, were removed from the data via linear regression. Detrending and filtering (0.01–0.08 Hz) were then sequentially performed.

ReHo analysis was performed on the preprocessed images described above. Individual ReHo maps were generated by calculating Kendall’s coefficient of concordance of the time series of a given voxel with those of its nearest neighbours (26 voxels) [21]. ReHo of each voxel was normalized to the global mean ReHo in order to reduce the effects of individual variations. Finally, a smoothing function with a Gaussian kernel of 4 × 4 × 4 mm (full width at half maximum) was applied.

### Statistical Analysis

Differences in demographic characteristics and cognitive scores between the two stroke groups (PSGC and PSPC) and the HC group were assessed using one-way analysis of variance (ANOVA) for continuous variables and the *X* ^2^ test for proportions using the SPSS Statistics 17.0 software package. The threshold for statistical significance was set at *P* < 0.05.

For within-group analysis, one-sample *t*-tests were performed to identify the ReHo patterns of the two stroke groups and the HC group using the Statistical Analysis tool of REST. The mean ReHo of each voxel in each group was compared with scalar 1. The threshold for significance was set to *P* < 0.01, and *P* values were corrected using the false discovery rate criterion.

A one-way ANOVA with age, sex and education years as covariates was performed to identify brain regions in which the spontaneous activity pattern was different between the PSGC, PSPC and HC groups. The F statistic was calculated using the following formula [34, 35]:

F=[SSB/(k−1)]/[SSW/(N−k)]

Note that k = 3 in our study.

k represents the number of groups, and N represents the total number of observations. The sum of squares between (SSB) is the squared difference between the group means and the global mean (the overall mean of all observations), whereas the sum of the squares within (SSW) is the sum of the squared difference between individual observations in a given group and the group mean. The degrees of freedom between is k−1, and the degrees of freedom within is N−k.

The resultant F statistic map was then thresholded using a corrected *P* < 0.01 as determined by a Monte Carlo simulation (see AlphaSim in AFNI; http://afni.nimh.nih.gov/pub/dist/doc/manual/AlphaSim.pdf) using the following parameters: single voxel *P* < 0.05; re-estimated FWHM = 6 mm; cluster size of at least 66 voxels; and the sum aggregated ReHo templates from the results of each one-sample *t*-test of the PSGC, PSPC and HC groups as a mask. Subsequently, post hoc analysis using the two-sample *t*-test was performed to compare the ReHo indexes between the PSPC group and the other two groups (PSGC and HC groups). The significance threshold levels were set at *P* < 0.01, and the *P* values were corrected and determined by Monte Carlo simulations using the following parameters: *P* < 0.05 for a single voxel and a minimum cluster size of 18 mm^3^ (AFNI AlphaSim). Age, sex, education and the time post-stroke were treated as covariates to avoid the potential influences of these factors. Of note, the post hoc comparisons were performed only for those regions showing a significant difference in ReHo based on ANOVA.

To identify the relationship between each patient’s post-stroke neuropsychological performance and the ReHo index of the regions identified by one-way ANOVA, partial correlation analysis (with the statistical significance threshold set to *P* < 0.05) was performed using SPSS version 17.0. The Bonferroni correction was applied for multiple comparisons in the correlation analyses. The ReHo of the regions identified by one-way ANOVA were extracted using REST software. Subsequently, these ReHo results and the neuropsychological scores were input into SPSS software to perform a partial correlation analysis with age, sex, education and the time post-stroke as control variables. Finally, although we did not report statistical significance of the differences in the volume of ischaemic lesions, their sizes were homogeneous.

## Results

### Clinical Data and Neuropsychological Tests

**Fig 1:**
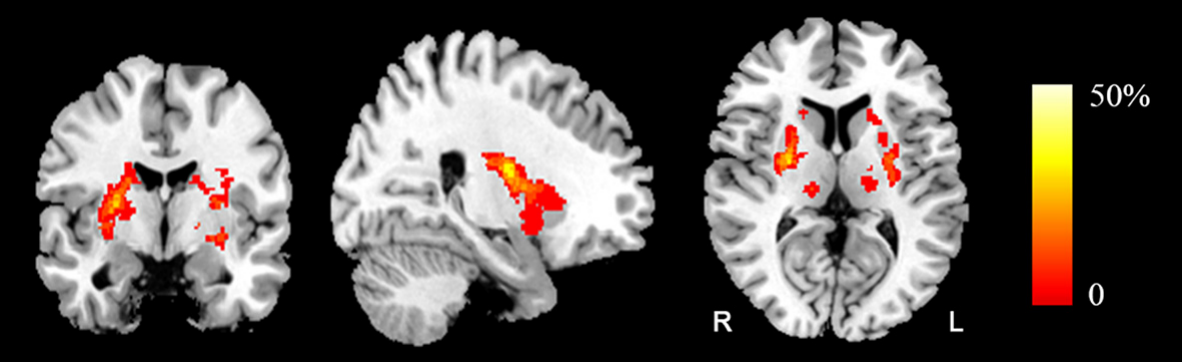
Lesion incidence map of patients with stroke. The coloured bar denotes the lesion incidence. R, right; L, left.

The PSGC, PSPC and HC (n = 30) groups did not significantly differ in age, sex, education or any vascular risk factor (all *P* > 0.05, Table 1). Comparing the PSGC (n = 16) and PSPC (n = 16) groups, no significant differences in the time post-stroke, lesion side or lesion volume were observed (all *P* > 0.05, Table 1). In 32 stroke patients (PSGC and PSPC), the subcortical ischaemic stroke lesions involved the internal capsule and surrounding structures, including the thalamus, basal ganglia and corona radiata (Fig 1); the infarcts were in the right hemisphere in 17 of these 32 patients and in the left hemisphere in the remaining 15 patients, and all infarcts were in the territory irrigated by the middle cerebral artery. The detailed neuroimaging characteristics of the two stroke groups (PSGC and PSPC) are listed in S1 Table. The PSGC and PSPC groups exhibited significantly worse neurocognitive performance than the HC group. Moreover, the PSPC group showed greater deficiency in several domains of cognitive performance than the PSGC group (Table 2, S1 Dataset).

**Table 1:** Demographic and clinical data of healthy control subjects and stroke patients. One-way analysis of variance (ANOVA) for continuous variables among the three groups. Chi-square test for categorical variables.

	HC	PSGC	PSPC	*P*
	(n = 30)	(n = 16)	(n = 16)	Value
**Demographic factors**				
Age, mean ± SD (y)	56.97±6.25	57.31±7.33	61.87±8.12	0.072
Age range (y)	48–69	45–72	48–75	—
Female	13(43.33%)	3(18.75%)	7(43.75%)	0.211
Education (years)	10.70±2.09	11.37±3.50	10.06±3.42	0.437
Lesion side (left/right)	—	7/9	8/8	0.723‡
Time post-stroke	—	13.81±8.49	11.56±8.56	0.461‡
Lesion volume (ml)	—	0.32±0.15	0.45±0.24	0.079‡
**Vascular risk factors**	—			
Hypertension	6(20.00%)	3(18.75%)	5(31.25%)	0.626
Dyslipidaemia	10(33.33%)	6(37.50%)	7(43.75%)	0.784
Diabetes	12(40.00%)	9(56.25%)	7(43.75%)	0.568
Smoking	13(43.33%)	6(37.50%)	8(50.00%)	0.775
Alcohol intake	9(30.00%)	5(31.25%)	6(37.50%)	0.870

‡ Indicates a significant difference between PSGC and PSPC based on the independent-samples t-test or the Chi-square test.

**Table 2:** Neuropsychological characteristics of healthy control subjects and stroke patients. Data are presented as means ± SD. MoCA: Montreal Cognitive Assessment; AVLT: Auditory Verbal Learning Test; TMT-A: Trail Making Test, Part A; TMT-B: Trail Making Test, Part B; CFT: Complex Figure Test; DST-F: Forward Digit Span Test; DST-B: Backward Digit Span Test; SDMT: Symbol Digit Modalities Test.

	HC	PSGC	PSPC	P
	(n = 30)	(n = 16)	(n = 16)	value*
**Cognitive performance**				
Mini Mental State Exam	28.30±1.44	26.94±1.29	24.93±2.40	< 0.001‡
MoCA	26.17±2.23	24.88±1.26	18.00±2.85	< 0.001‡
AVLT	6.64±2.15	5.98±1.31	4.77±1.16	0.004
AVLT-delayed recall (20 min)	6.60±1.87	5.19±2.10	2.81±2.54	< 0.001‡
TMT-A	59.80±15.93	48.69±12.95	72.56±25.36	0.002‡
TMT-B	139.43±41.99	159.88±60.51	255.13±115.59	< 0.001‡
CFT	35.28±1.42	35.38±1.41	31.88±6.54	0.006‡
CFT-delayed recall (20 min)	18.95±5.37	17.13±4.29	10.06±6.30	< 0.001‡
DST-F	7.57±1.63	7.44±1.15	6.13±1.41	0.007‡
DST-B	4.73±1.34	4.44±0.89	3.44±1.21	0.004‡
SDMT	34.10±8.76	33.75±6.27	23.81±10.63	0.003‡

* Indicates a significant difference between the three groups based on one-way analysis of variance (ANOVA).

‡ Indicates significant differences in the neuropsychological test results between the PSGC and PSPC groups (*P* < 0.05).

### ReHo Analysis

#### Within-Group ReHo Analysis

**Fig 2:**
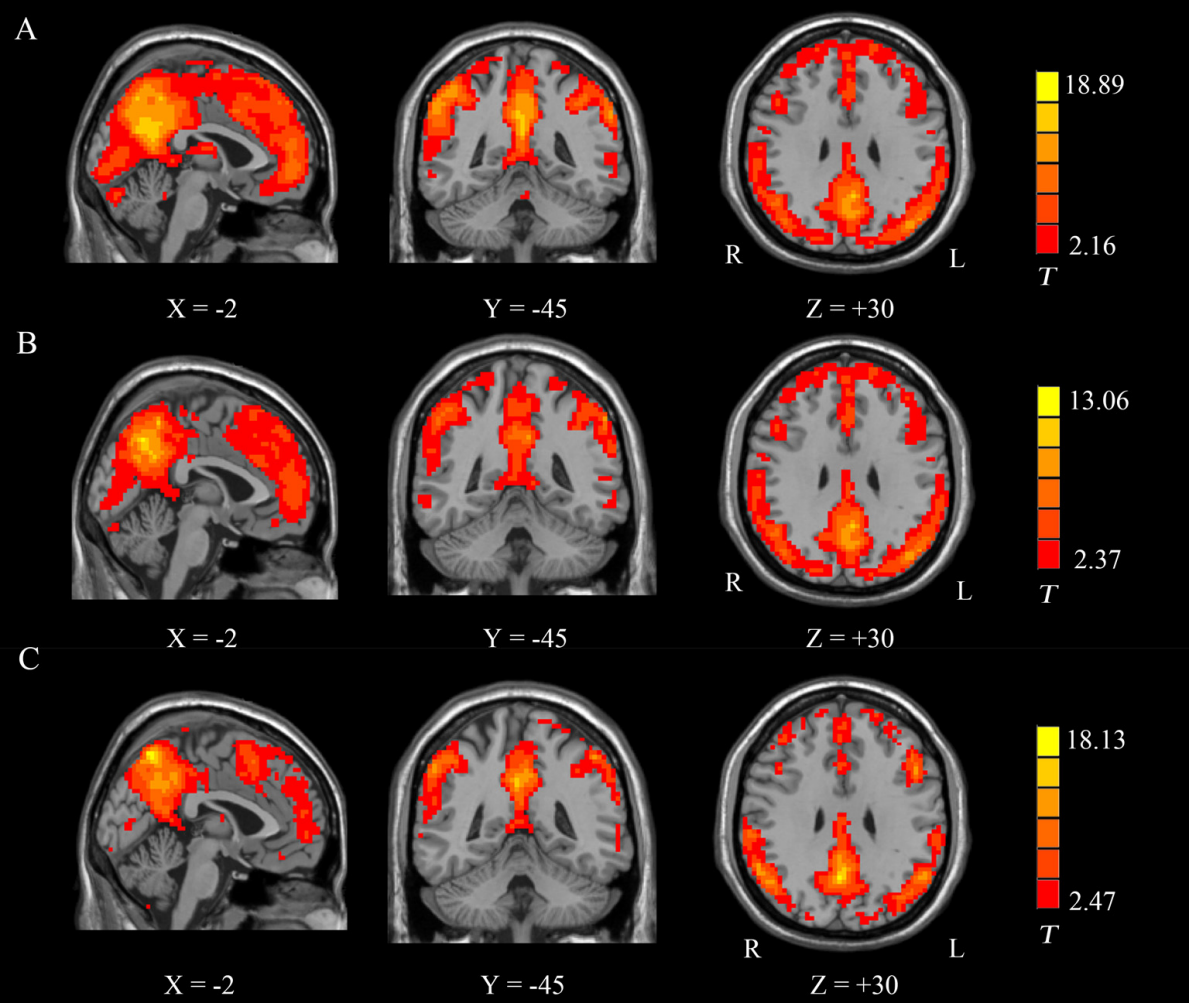
Group-level analyses of the ReHo patterns. (A) HC: healthy controls; (B) PSGC: stroke patients with good cognitive function; (C) PSPC: stroke patients with poor cognitive function. *T* = results from one-sample tests; X, Y, and Z = x, y, and z Montreal Neurological Institute (MNI) coordinates, respectively. R, right; L, left.

The ReHo maps of all three groups are presented in Fig 2. The standardized ReHo in the PCC/precuneus, the ACC, the medial prefrontal cortex, the inferior parietal lobule and the lateral temporal lobe were significantly higher than the global mean ReHo in each group.

#### One-way ANOVA and Post Hoc ReHo Analysis

**Fig 3:**
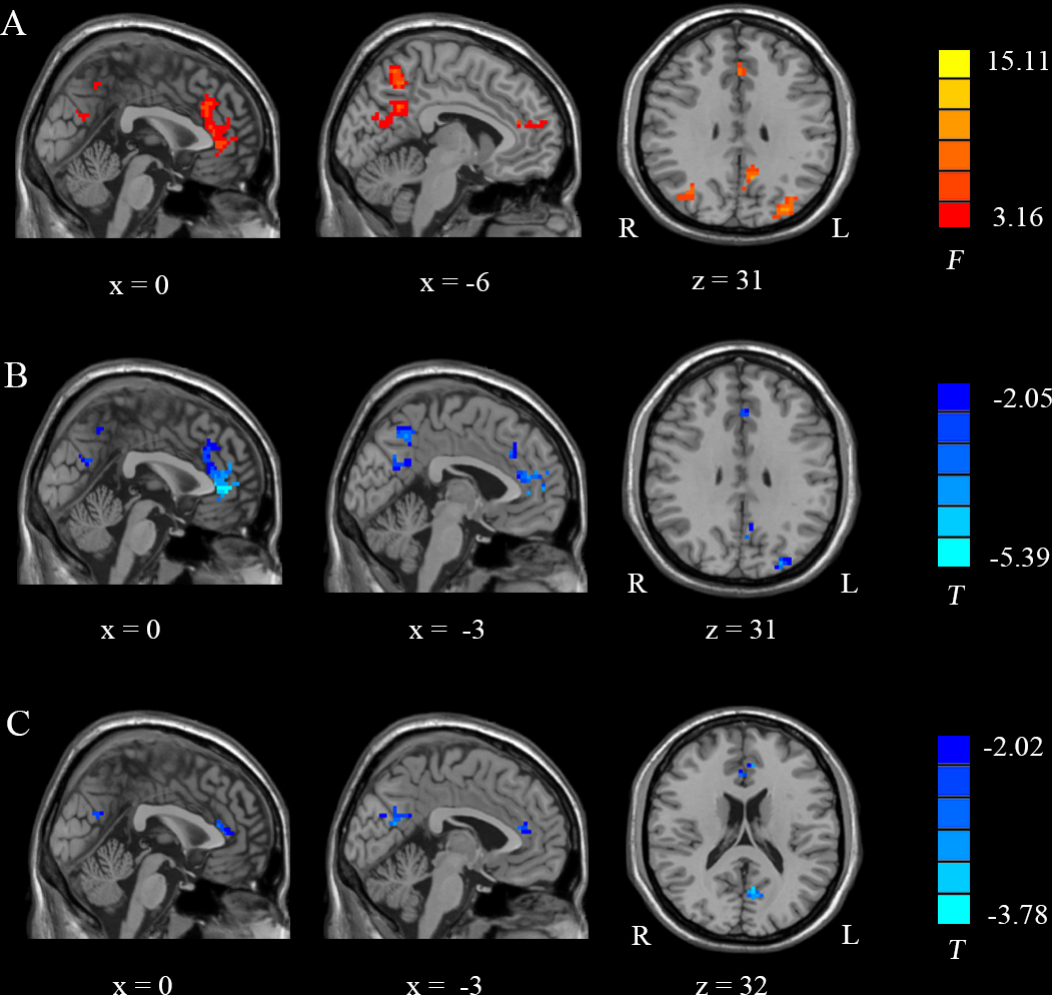
One-way ANOVA and post hoc ReHo analysis results. (A): Detailed regions in the images show differences in ReHo within the DMN between the three groups. (B) and (C): Detailed regions in the images show decreased ReHo in (B) PSPC patients compared to PSGC patients and (C) PSPC patients compared to HC. *F* = results from ANOVA; *T* = results from two-sample *t*-test; X and Z = x and z Montreal Neurological Institute (MNI) coordinates, respectively; R, right; L, left.

Fig 3A and Table 3 show the results of a one-way ANOVA between the three groups. Several brain regions, including the bilateral ACC, the left PCC/precuneus and the bilateral occipital lobes, displayed significant differences in ReHo.

**Table 3:** Brain regions showing significantly different ReHo between the two groups of post subcortical stroke patients and the healthy control group (ANOVA). Comparisons were performed at P < 0.01, AlphaSim-corrected; MNI, Montreal Neurological Institute; R, right; L, ACC: Anterior cingulate cortex; PCC: Posterior cingulate cortex.

Brain region	Brodmann’s Area	Peak MNI			Volume	F value of	Maximum
		coordinates			(mm^3^)	peak voxel	Z score
		X	Y	Z			
Bilateral ACC	32 and 10	0	30	30	119	7.10	1.62
L PCC/ precuneus	31	-9	-57	30	76	7.66	1.87
L Occipital lobe	19	-33	-87	24	115	15.11	1.59
R Occipital lobe	7	24	-66	39	68	8.30	1.54

Fig 3B–3C and Table 4 show the results of post hoc comparisons using a two-sample *t*-test between the PSPC group and the other two groups (PSGC and HC groups). Compared with the PSGC group, the PSPC group showed remarkably decreased ReHo in the bilateral ACC, the left PCC/precuneus and the left occipital lobes. Compared to the HC group, the PSPC group showed significantly reduced ReHo in the ACC and the left PCC/precuneus.

**Table 4:** Results of post hoc analyses based on two-sample *t*-tests between the PSPC group and the other two groups (PSGC and HC). ACC: Anterior cingulate cortex. Comparisons were performed using a significance threshold of P < 0.01, AlphaSim-corrected; MNI, Montreal Neurological Institute; R, right; L, left.

Group comparison and brain region	Brodmann’s area	Peak MNI coordinates			Volume (mm^3^)	T value of peak voxel
		X	Y	Z		
**PSPC < PSGC**						
Bilateral ACC	32 and 10	0	45	3	112	-5.39
L PCC/ precuneus	31	-3	-72	27	39	-3.03
L occipital lobe	19	-27	-87	30	71	-4.22
**PSPC < HC**						
Bilateral ACC	32	-6	39	18	25	-3.19
L PCC/ precuneus	31	-6	-66	18	38	-3.30

### Correlation Analysis

**Fig 4:**
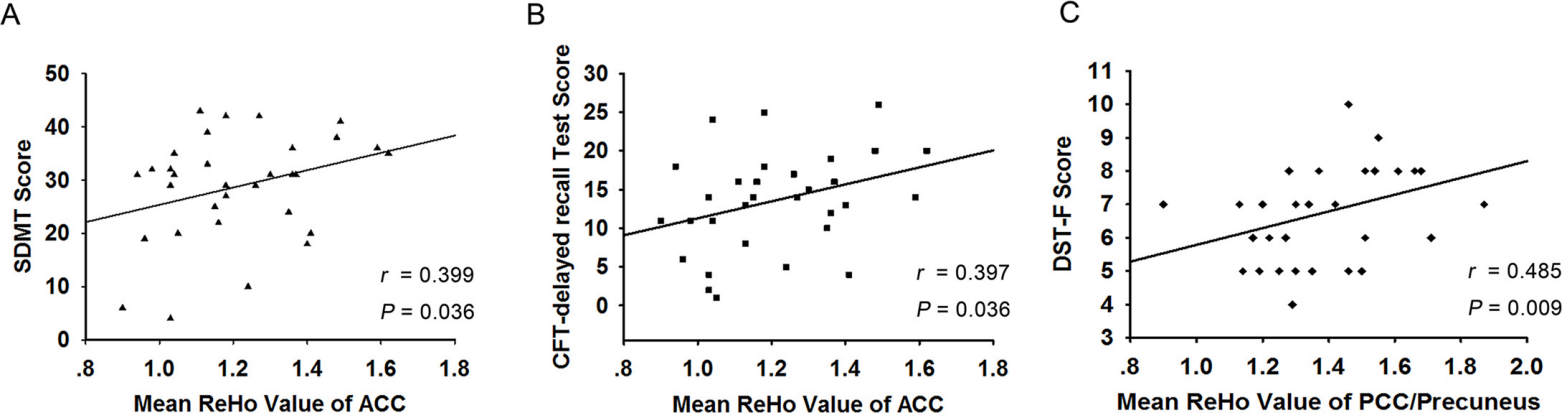
Scatterplot showing the significant positive correlations between the neuropsychiatric data and the ReHo indexes of the ACC and PCC/precuneus in all stroke patients. (A) The ReHo of the bilateral ACC positively correlated with the SDMT scores and (B) CFT-delay recall subtest scores in all stroke patients. (C) The ReHo of the left PCC/precuneus positively correlated with the DST-F test scores in all stroke patients.

The partial correlation results indicated that the ReHo of the bilateral ACC positively correlated with the SDMT and CFT-delay recall subtest scores (*r* = 0.399, *P* = 0.036; *r* = 0.397, *P* = 0.036) in all post-stroke patients, including both the PSGC and PSPC groups (Fig 4A and 4B). Moreover, the ReHo of the left PCC/precuneus positively correlated with the DST-F test scores (*r* = 0.485, *P* = 0.009) in all post-stroke patients (Fig 4C). No significant correlations were detected between cognitive performance and the ReHo of the bilateral occipital lobes. Nevertheless, no significant correlations persisted after Bonferroni correction.

## Discussion

The current study used the ReHo algorithm to demonstrate aberrant regional coherence in post-stroke patients. Compared with the PSGC and HC groups, the PSPC group displayed decreased ReHo in the bilateral ACC, the left PCC/precuneus and the occipital lobe. Moreover, aberrant ReHo correlated with impaired cognitive performance in all post-stroke patients. Thus, these findings may contribute to a better understanding of the neuro-pathophysiological mechanisms of cognitive dysfunction in post-stroke patients.

Although the neural mechanisms underlying the DMN disruptions observed in patients with stroke are uncertain, several candidates should be considered. After stroke, there are disruptions in the direct or indirect anatomical connections between the lesion and DMN regions such as the cingulate cortex [36]. Furthermore, subcortical vascular disease may contribute to the abnormal release of neurotransmitters and the deposition of amyloid β-protein within the DMN [37], an important resting-state functional network [38] that has been shown to be associated with cognitive and emotional processing in post-stroke patients [39, 40].

Previous studies [15, 16, 20] have focused on the FC within the DMN of stroke patients, showing decreased FC between the PCC and the ACC. In addition, patients with cognitive impairment had significantly decreased FC compared to those without cognitive impairment, whereas patients without cognitive impairment tended to have decreased FC compared to the HC [20]. ReHo reflects the changes in temporal aspects of spontaneous neuronal activity in a brain region [21]. Therefore, altered ReHo revealed the local destruction of spontaneous neuronal activity in certain regions, implying functional deficits [41]. In our study, decreased ReHo in the PCC and ACC supported the notion of DMN impairment in post-stroke patients. The FC of the DMN was reduced and spontaneous neuronal activity itself was weak in post-stroke patients with cognitive dysfunction.

Cognitive impairment after stroke involves numerous domains, most commonly including the decline of memory, attention and executive functions together with reduced reaction and information processing speeds [3, 42]. The ACC and the PCC belong to the limbic system, which greatly impacts the process of cognition [43, 44]. The ACC has occupied a central role in theories of attention and cognitive control, which hold that the ACC either monitors response conflict and signals the need for adjustments in cognitive processes or directly mediates such adjustments [44]. The PCC is a highly connected and metabolically active brain region within the DMN [43]. In addition to its involvement in the DMN, the PCC is involved in the dorsal attention network by exerting top-down control of visual attention as well as the frontoparietal control network, which is involved in executive motor control [43]. Many circuits, such as the cortical-striatal-thalamic-cortical circuit, exist in the human brain; lesions near the striatum, such as those observed in the current study, destroy the structures and functions of these circuits, thus affecting other brain areas and functional networks related to these circuits [45, 46]. Therefore, the lower ReHo indexes of the ACC and the PCC (specifically, the low consistency in neural activity) may reflect the impairment of the DMN and other resting-state networks, such as the attention and executive networks. We also observed that compared with the PSGC group, the PSPC group showed decreased ReHo in the bilateral occipital lobes. The occipital lobe is a component of the visual attention network. Therefore, patients with poor recovery of cognitive performance might exhibit more severe dysfunction in the visual attention network.

Based on correlation analysis, we found that in all post-stroke patients, decreased ReHo in the bilateral ACC positively correlated with the SDMT and CFT-delay recall subtest scores and that ReHo in the left PCC/precuneus positively correlated with the DST-F test scores. As mentioned above, performance on the SDMT, which is used to evaluate post-stroke patients [47], is underpinned by attention and perceptual speed [32]. DST-F [31] was also carried out to assess attention abilities. The ACC and PCC are related to the attention network, and key components of cognitive impairment after stroke include weakened attention and execution capabilities, such as information processing speed [3, 42]. Therefore, this correlation between the regional coherence alterations and the neuropsychological test scores corroborates these parameters. The decreased neuronal activity revealed by the ReHo index in the ACC and the PCC/precuneus suggested more severe cognitive impairments, whereas increased ReHo in the ACC and the PCC/precuneus indicated relatively good cognitive function.

Liu et al. [20] revealed that post-stroke patients exhibited decreased ReHo in the PCC and reduced FC between the PCC and the ACC. However, because only a global cognitive evaluation (MoCA) was used to assess cognitive function in Liu’s study, associations between ReHo alterations and specific cognitive domains, such as attention or memory function, could not be determined in stroke patients. Moreover, whether the decreased ReHo resulted from the stroke itself or from the differences in cognitive performance remained unclear. To address this issue, we set up three groups (PSPC, PSGC and HC) instead of only two groups (post-stroke patients and HC). The three-group design of this comparative study specifically revealed the synchronicity of neural activity resulting from different cognitive performances, eliminating the influence of the factors dependent on stroke itself because both the PSPC and PSGC groups consist of post-stroke patients. Moreover, the results of the comparison between the PSPC and HC groups confirmed the findings in the ACC and the left PCC/precuneus of the PSPC group. This experimental design helped to reveal the neurophysiological mechanisms of cognitive dysfunction after stroke.

Several limitations of this study should be acknowledged. First, the cross-sectional study design did not allow for the identification of dynamic changes in ReHo patterns after stroke. Second, the sample size of each group was relatively small. Therefore, replication of these findings in a longitudinal study with a larger sample size is required to confirm our results. Thirdly, the significant clusters in our study seemed too expanded, mixing grey and white matter. The 2dReHo method [48] which could perform calculations on the grey matter surface may be applied to resolve this question in the future. Moreover, the subdivision of the subcortical stroke group into unilateral or bilateral stroke groups would more clearly reveal the relationship between functional reorganization and cognitive impairment. Finally, although multiple cognitive scales have been used in this study, the establishment of a neuropsychological scale as a gold standard remains necessary to obtain a more specific assessment of post-stroke cognitive dysfunction in future studies.

## Conclusion

In summary, compared to the PSGC and HC groups, the PSPC group demonstrated altered regional coherence, primarily within the cingulate cortex. We also identified a correlation between deficient cognitive performance and decreased ReHo in the ACC and PCC/precuneus. These findings support the use of ReHo as a promising indicator that can provide essential insights into the neuro-pathophysiological mechanisms of post-stroke cognitive dysfunction.

## Supporting Information

S1 Table Neuroimaging characteristics of the two stroke groups (PSGC and PSPC). (DOCX)

S1 Dataset The detailed clinical information for all subjects. (SAV)
