# Supplementary material for: Regional Coherence Alterations Revealed by Resting-State fMRI in Post-Stroke Patients with Cognitive Dysfunction
Source: PLoS One. 2016 Jul 25;11(7):e0159574. doi: 10.1371/journal.pone.0159574 (PMC4959733; doi:10.1371/journal.pone.0159574)
Supplement: S1 Table — (DOCX) [file pone.0159574.s001.docx]

Table S1: Neuroimaging characteristics of the two stroke groups (PSGC and PSPC)

| Group | Side  of lesion | Vascular distribution | Infarct location | Months  since  stroke | Lesion  volume  (ml) |
| --- | --- | --- | --- | --- | --- |
| PSGC |  |  |  |  |  |
| 1 | R | MCA | BG | 21.00 | 0.24 |
| 2 | L | MCA | BG | 12.00 | 0.26 |
| 3 | R | MCA | TH | 5.00 | 0.19 |
| 4 | R | MCA | IC | 22.00 | 0.26 |
| 5 | R | MCA | CR | 12.00 | 0.23 |
| 6 | L | MCA | TH | 14.00 | 0.20 |
| 7 | R | MCA | IC | 7.00 | 0.55 |
| 8 | R | MCA | TH | 3.00 | 0.68 |
| 9 | L | MCA | BG | 21.00 | 0.24 |
| 10 | L | MCA | BG | 18.00 | 0.34 |
| 11 | L | MCA | BG | 20.00 | 0.24 |
| 12 | R | MCA | BG | 3.00 | 0.40 |
| 13 | R | MCA | TH | 24.00 | 0.24 |
| 14 | L | MCA | BG | 29.00 | 0.14 |
| 15 | L | MCA | BG | 5.00 | 0.48 |
| 16 | R | MCA | TH | 5.00 | 0.39 |
| PSPC |  |  |  |  |  |
| 1 | R | MCA | BG | 4.00 | 0.72 |
| 2 | L | MCA | TH | 6.00 | 0.31 |
| 3 | R | MCA | IC | 8.00 | 0.78 |
| 4 | L | MCA | CR+BG | 24.00 | 0.90 |
| 5 | L | MCA | IC | 21.00 | 0.46 |
| 6 | L | MCA | CR | 20.00 | 0.19 |
| 7 | R | MCA | BG | 18.00 | 0.36 |
| 8 | R | MCA | BG | 25.00 | 0.36 |
| 9 | L | MCA | BG | 24.00 | 0.04 |
| 10 | L | MCA | TH | 5.00 | 0.39 |
| 11 | R | MCA | TH+BG | 5.00 | 0.77 |
| 12 | R | MCA | TH+CR | 3.00 | 0.23 |
| 13 | R | MCA | TH+CR | 6.00 | 0.32 |
| 14 | L | MCA | BG+CR | 5.00 | 0.53 |
| 15 | R | MCA | BG | 5.00 | 0.52 |
| 16 | L | MCA | TH | 6.00 | 0.27 |

BG: basal ganglia; TH: Thalamus; IC: intern capsule; CR: corona radiate; MCA: middle cerebral artery; L: left; R: right.
